# Evaluation Model Based on the SGCNiFormer for the Influence of Different Storage Environments on Wheat Quality

**DOI:** 10.3390/foods14101715

**Published:** 2025-05-12

**Authors:** Qingchuan Zhang, Zexi Song, Mingwen Bi

**Affiliations:** 1 National Engineering Research Centre for Agri-Product Quality Traceability, Beijing Technology and Business University, No. 11 and No. 33, Fucheng Road, Haidian District, Beijing 100048, China; zhangqingchuan@btbu.edu.cn; 2 Business School, Beijing Wuzi University, No. 321, Fuhe Street, Tongzhou District, Beijing 101149, China

**Keywords:** predicting changes in wheat quality, wheat storage, wheat, prediction, GCN

## Abstract

Wheat is a vital staple food crop, and its post-harvest storage is paramount to maintaining its quality. However, conventional grain storage methods frequently impede the ability to promptly and accurately predict and assess quality changes. Moreover, most storage systems are ineffective in dealing with the impact of temperature and humidity fluctuations on wheat quality, which can potentially lead to quality degradation during storage. To address these challenges, this paper proposes a dual model system of “prediction-evaluation”, which integrates a dynamic quality prediction model based on SGCNiFormer with an evaluation framework based on K-Smeans clustering to establish a closed-loop mechanism from quality prediction to storage effect evaluation. The system incorporates a graph convolutional network (GCN) and a dynamic gating module, enabling precise simulation of the multidimensional evolution of wheat quality under the interaction of moisture and temperature. The experimental results demonstrate the superiority of SGCNiFormer in time-series prediction tasks, while the K-Smeans method establishes a wheat quality grading standard with physical interpretability. This integrated method provides a systematic theoretical framework for optimizing storage parameters and offers substantial support for intelligent grain storage management.

## 1. Introduction

China is the world’s leading producer and exporter of wheat, which is the staple food for approximately 40% of the global population and plays a pivotal role in the Chinese diet. Wheat is rich in nutrients [1], including starch, protein, fat, minerals, calcium, iron, thiamine, riboflavin, niacin, vitamin A, and vitamin C [2]. However, fluctuations in environmental parameters during storage can lead to nutrient loss [3]. Consequently, the establishment of an optimal storage environment emerges as a pivotal strategy to ensure the preservation of quality. Empirical evidence has demonstrated that effective wheat storage conditions are characterized by low temperatures, minimal humidity, and adequate ventilation. These conditions impede the occurrence of enzymatic reactions, reduce lipid oxidation [4], and hinder microbial metabolism, thereby sustaining the physiological activity of wheat. When the storage temperature is too high, respiration and the rate of fatty acid rancidity increase significantly, while high humidity can lead to the rapid spread of mold [5], causing the accumulation of harmful substances such as aflatoxins. Therefore, precisely controlling the temperature and humidity gradient and their coupling effect has become the core proposition for balancing storage safety and economy.

This study takes a multidimensional approach to the quality deterioration process and establishes a three-in-one evaluation system for physiological activity, chemical stability, and physical characteristics. The germination rate is used as an indicator of the physiological and metabolic strength of the seed, the fatty acid value is used to monitor lipid oxidation, and the weight capacity index is introduced to quantify the physical basis of processing performance. These three key parameters correspond to the cell-level viability maintenance ability, molecular-level component stability, and macro-level process adaptability of wheat during storage, and together they form an early warning signal network for quality deterioration.

During storage, the physiological state of wheat is a key factor affecting its quality. Wheat germination rate [5,6] is an important indicator of seed vitality, which directly reflects the health of the seed during storage [7]. In addition to physiological indicators, chemical indicators of wheat are also an important basis for predicting storage quality. The fatty acid value is a measure of the degree of lipid oxidation in wheat, which is significantly affected by storage temperature and humidity. Lipid oxidation not only causes off-flavors in wheat but also reduces its processing suitability and affects the sensory quality of the final food product. The indicator of the physical property dimension, the test weight, is directly related to the compactness of the endosperm structure and the efficiency of flour extraction, providing a quantitative basis for processing suitability. Ivica Strelec et al. [8] continuously monitored the dynamic changes in multiple quality indicators of three wheat varieties during a 12-month storage period under different storage temperature and humidity conditions and drew important conclusions based on the results.

With the development of human society, people around the world are paying more and more attention to the quality of food every year, and how to protect the quality of food during storage has become one of the important issues. Different storage environments are crucial for the protection of food quality. Therefore, choosing the storage environment for food storage is one of the urgent problems that needs to be solved. Clare Kyomugasho et al. [9] established a link between the relative position of the storage conditions of beans along the Tg line (their glass transition temperatures) and the degree of hard cooking (HTC) development. The rate of change in cooking time with storage time during storage was thus obtained. Coradi, P.C. et al. [10] explored the seed quality of different varieties of soybean seeds after 6 months of storage in different packages and concluded that the technical laminated packaging is more advantageous. Wawrzyniak, J. et al. [11] developed a model to predict the impact of mycotoxins in stored barley, which can be used to estimate the severity of fungal contamination in large quantities. Mohamed Nejib El Melki et al. [12] constructed a multi-parameter storage simulation system to systematically monitor the population changes in the Dominican wheat midge (*Sitophilus dominicanus*) under gradient temperature and humidity and ventilation intensity. The research revealed the development and reproduction threshold of this storage pest, established a mapping relationship between the three-dimensional distribution of temperature and humidity and the pest index, and ultimately formed a pest suppression plan based on environmental regulation.

Researchers have found that time-series analysis, as a key technique for revealing the evolution of systems, has evolved from traditional statistical modeling to a research system that integrates multiple methods. Classical statistical models [13,14] construct a stable sequence prediction framework through differential operations and linear combinations, maintaining theoretical advantages in scenarios that satisfy the Gaussian assumption. However, in the face of the nonlinear characteristics and time-varying covariance structure in complex systems, support vector regression, gradient boosted trees, and other methods based on machine learning [15,16] have effectively improved modeling capabilities through feature engineering. In recent years, deep learning architectures [17,18,19] have made breakthroughs in capturing long-term dependencies with their multi-level feature extraction mechanisms. The research paradigm has shifted from single model optimization to hybrid modeling strategies, with a focus on the physical interpretability of models and an in-depth analysis of the coupling mechanisms of dynamic systems, marking a new stage in time-series analysis, with the integration of multi-scale features [20,21]. Han, H.M. et al. [22] proposed an EEG-GCN architecture for predicting time-series data, utilizing GCN to identify feature interactions between sub-signals and map the interdependencies among data. Cheng, J.D. et al. [23] proposed a tensor form to comprehensively describe the information of time-series PolSAR data (time-series polarimetric synthetic aperture radar, an effective technique for crop classification and agricultural activity monitoring), including spatial context information, polarization scattering information, and temporal context information. Geovane da Silva André et al. [24] comparing the performance parameters of four types of predictive models (ANN, REPTree, M5P, RF and LR), it was found that the prediction errors of the random forest (RF) and the rapid decision tree (REPTree) for the physical integrity (damage rate, moisture content) and physiological potential (enzyme activity, germination potential) of soybean seeds were significantly lower than those of the traditional linear regression model, which verified the advantages of tree structure algorithms in modeling nonlinear agricultural data. Lance E.R. et al. [25] investigated the trend of wheat moisture content over storage time and used an ARIMA model to predict it. Koomson P. et al. [26] used an LSTM model to predict grain temperature based on past collected data to predict stored grain temperature, thereby avoiding deterioration of the grain during storage. Uma Kamboj et al. [27] studied the quality parameters of wheat grains stored for one year. Principal component analysis (PCA) scores and other methods were used to analyze the reference data and near-infrared spectroscopy data. All models were able to accurately predict the protein and carbohydrate content of stored wheat, with PLS and SVM providing the best results. Liang Ge et al. [28] proposed a CLSTM model, which was used to predict the temperature inside the grain pile, thereby reducing grain loss and improving quality.

In recent years, clustering algorithms have been applied to time-series analysis. Wang, R. et al. [29] proposed the i-MFEA time-series clustering algorithm based on evolutionary multi-task optimization, which uses an improved multi-factor evolutionary algorithm to simultaneously optimize multiple clustering tasks. Lee, J. et al. [30] proposed the TMRC model to address time-series clustering. By extracting features from different temporal patterns using TMRL, these features are then utilized for time-series clustering.

It is different from the existing models that only use time-series analysis models [31] to predict some indicators of crops during the storage process, thereby screening out the environment more suitable for storing crops, or only use related models, such as clustering, to classify the indicators of crops by grade. This study aims to investigate a complete dual-model system for wheat storage and grade classification. This is not merely a simple combination of the two models. Instead, an efficient time-series prediction model is established first, and an accurate clustering model is designed based on the time-series prediction results. These steps are interlinked to form a dual-model system for wheat quality prediction and evaluation. Provide theoretical support and technical guidance for the storage management and quality improvement of wheat.

The present study focuses on the multidimensional evolution of wheat quality during storage. By integrating time-series prediction and dynamic clustering methods, the collaborative degradation mechanism of physiological activity, chemical stability, and physical properties is systematically analyzed. In response to the need for dynamic quality prediction under the coupled action of temperature and moisture content, a multimodal time-series prediction framework based on the SGCNiFormer model is proposed. The model incorporates a graph convolutional network (GCN) to establish the topological correlation between the environment and quality indicators, and it introduces a dynamic gating mechanism to integrate time-series features and spatial dependencies. The development of a K-Smeans clustering algorithm that incorporates time-series information was based on these innovations. The construction of a spatiotemporal feature matrix integrates historical observations and future predictions, and this matrix is then used to classify storage quality levels. Experimental findings demonstrate that the SGCNiFormer model enhances the prediction accuracy of key indicators, while the K-Smeans clustering algorithm enhances the clustering evaluation indicators in comparison to traditional K-means, effectively classifying wheat quality into five interpretable levels. This dual-model system differs from existing models in that it can more accurately predict wheat quality evaluation indicators while utilizing time-series data and clustering algorithms to classify wheat quality indicators. This enables not only the prediction of future data for quality indicators of different wheat varieties under different storage conditions, but also the prediction of changes in quality grades caused by storage. This provides a quantitative decision-making basis for precise control of storage parameters, significantly enhancing the predictability and scientific nature of storage management.

The primary contributions of this study are as follows:

- The GCN-based temporal model SGCNiFormer, which has been demonstrated to outperform mainstream models in the context of long-term multivariate temporal prediction, achieving a 58% reduction in MAE;
- A dynamic clustering framework, K-Smeans, integrating temporal dependencies to classify wheat quality into five interpretable grades, which has been validated to outperform K-means clustering after evaluation using clustering metrics such as the contour coefficient;
- Establishing a “prediction-evaluation” dual-model system based on SGCNiFormer and K-means, providing a quantifiable decision-making basis for intelligent grain warehouse management.

## 2. Materials and Methods

### 2.1. Materials

#### 2.1.1. Data Sources

**Figure 1:**
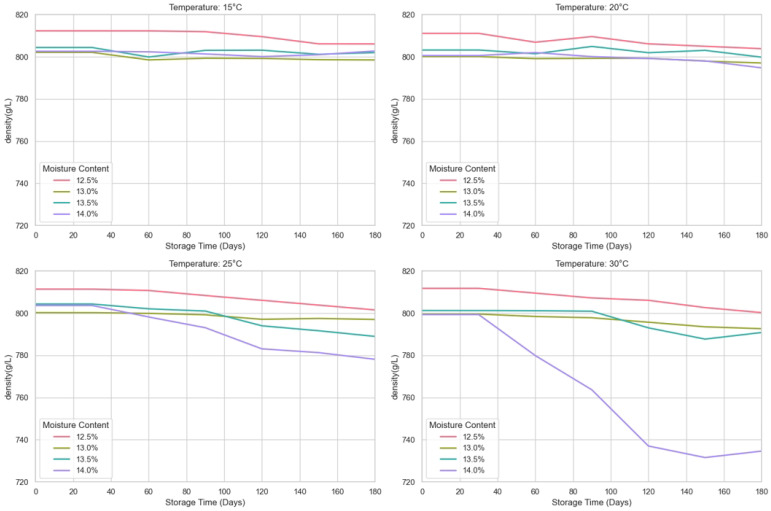
Sampling dataset for wheat test weight.

**Figure 2:**
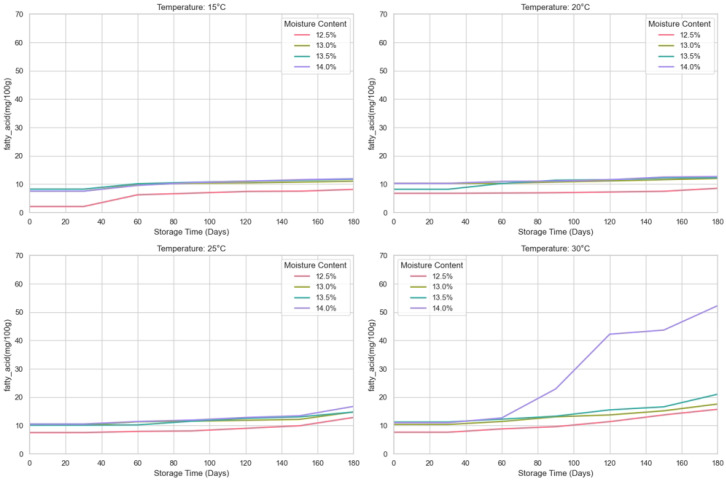
Sampled dataset of the fatty acid value of wheat.

**Figure 3:**
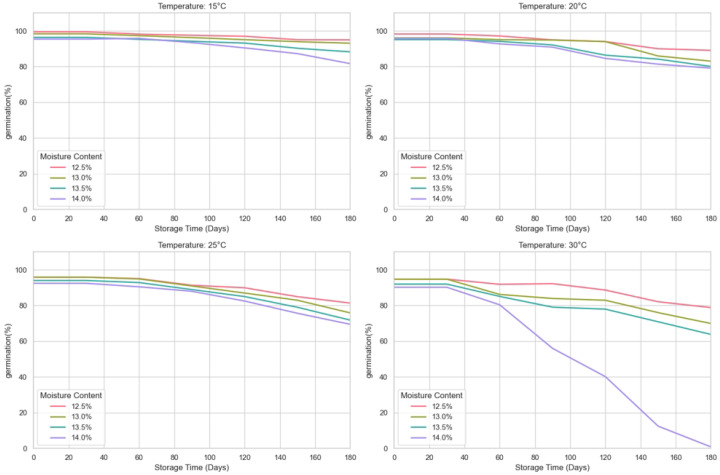
Sampling dataset of wheat germination rate.

This study investigates the effects of storage temperature and moisture on the storage quality of wheat by predicting different wheat quality evaluation indicators during storage at different temperatures (15 °C, 20 °C, 25 °C, 30 °C) for wheat with moisture contents of 12.5%, 13%, 13.5%, and 14%. The indicators of wheat quality are germination rate, electrical conductivity, fatty acid value, gluten water absorption rate, and final viscosity. The experiment was set up as follows: the experiment was carried out in a constant temperature and humidity chamber. According to the methods proposed by Lutz and Coradi [32], the moisture content of the wheat was calibrated to the levels of 12.5 ± 0.1%, 13 ± 0.1%, 13.5 ± 0.1%, and 14 ± 0.1%. The wheat samples were then stored for 180 days under different temperature conditions. In addition, the temperature of the constant temperature and humidity box was set according to the principle of equilibrium moisture, as shown in Table 1, to ensure stable conditions for storing the wheat. Throughout the storage period, wheat samples were collected regularly every 30 days to analyze the relevant indicators. The germination rate of wheat during storage was determined according to GB/T 5520-2011 (China), the bulk density was determined according to GB/T 5498-2013 (China), and the method of Zhang and Liu [33] was used to determine the fatty acid value with slight modifications. The wheat sample was ground into a fine powder using a hammer mill. Fatty acids were extracted from 10 g of wheat flour using benzene as the solvent, with a 0.04% phenolphthalein solution as the indicator, and finally titrated with 0.01 mol/L KOH. The result is expressed in mg/100 g. We have transformed the collected data receipts into line graphs as shown in Figure 1, Figure 2 and Figure 3.

The wheat storage quality dataset constructed in this study contains 560 observed samples and is composed of full-factor experiments with 4 moisture gradients, 4 temperature conditions, and 7 time nodes. Each treatment combination conducts 5 technical repeat measurements at each sampling time, and a total of data records of three core quality indicators, namely germination rate, fatty acid value, and bulk density, are collected. The data are stored in a long format, with each sample labeled with moisture, temperature, time, and repeated numbering. It supports multi-factor interaction effect analysis and time-series modeling. Meanwhile, it quantifies experimental errors through repeated measurements, providing a high-resolution data basis for evaluating the dynamic impact of storage conditions. Subsequently, systematic preprocessing was carried out on the obtained spatio-temporal multi-dimensional quality data. For the dimensional differences of germination rate, fatty acid value, and bulk density, standardization processing was implemented, respectively, based on the distribution characteristics of the indicators, including range transformation and normalization transformation. For 1.2% of the randomly missing data, a method combining time-series prediction and multi-dimensional parameter space interpolation is adopted for repair, and abnormal observations are eliminated through statistical tests. This processing flow effectively reduces the interference of dimensional differences and data omissions on model training while retaining the biological significance of the indicators. The input format of the SGCNiFormer model proposed by us includes the time column, the storage temperature column, the wheat moisture content column, the germination rate column, the fatty acid column, and the bulk density column.

#### 2.1.2. Experimental Environment

In this study, the SGCNiFormer model was constructed using the deep learning framework PyTorch(2.0.0+cu118) [34], a Python3.9-based scientific computing library that provides highly flexible deep learning tools and supports both dynamic and static computational graphs.

### 2.2. Proposed Model

**Figure 4:**
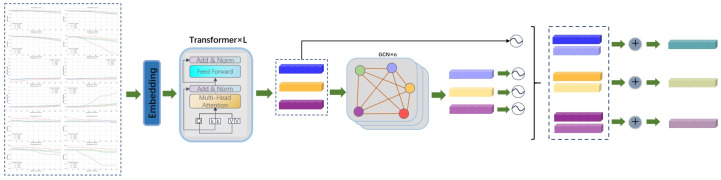
Flowchart of SGCNiFormer model operation.

Despite the notable success of transformer-based methods in addressing multivariate time-series prediction, a notable gap remains in the field concerning the analysis of dependence between multivariate series. To address these issues, this paper proposes SGCNiFormer, a framework that is a sequence-aware graph convolutional network Transformer model. It combines the advantages of iTransformer for long-term multivariate time-series prediction and designs a GCN module to efficiently capture and analyze the dependencies between sequences, as shown in Figure 4. The efficacy of SGCNiFormer in addressing the limitations of conventional Transformer-based methods in handling multivariate sequence dependencies is demonstrated by its ability to effectively capture and analyze the dependencies between long-term multivariate time series. The findings indicate that SGCNiFormer has attained substantial advantages in terms of evaluation metrics such as root mean square error (RMSE) and mean absolute error (MAE) and can more accurately predict long-term multivariate time series. This conclusively demonstrates its remarkable performance in dealing with multivariate sequence dependencies and long-term prediction.

In this model architecture, the processing of multivariate time series refers to the design of iTransformer[], which processes different variables. For the multivariate time-series data $X=x_{1},\ldots ,x_{T}\in R^{T\times N\;}$ of the input wheat quality index, the data are processed through the embedding layer.

H=Embedding(X)

Among them, Embedding· is the embedding layer processing, and $H=h_{1},\ldots ,h_{N}\in R^{N\times D}$ is the result after processing. When calculating attention, the query (*Q*), key (*K*), and value (*V*) are obtained by linear projection, and the calculation process is as follows:

$$Q=HW_{Q},K=HW_{K},V=HW_{V}$$

where $W_{Q}$, $W_{K}$, $W_{V}$ are learnable projection matrices and $Q,K,V\in R^{N\times d_{k}}$ and $d_{k}$ are the projection dimensions. After processing by the softmax function, the weighted attention weights are obtained, and the attention-weighted attention calculation is calculated as follows:

$$Attention\left(H\right)=softmax\left(\frac{QK^{⊤}}{\sqrt{d_{k}}}\right)V$$

The feedforward neural network (FFN) within the Transformer is responsible for additional feature transformation and encoding of the variable tokens that are processed by the attention mechanism. The calculation formula for the FFN is as follows:

$$FFN\left(h_{n}\right)=\max\left(0,W_{1}h_{n}+b_{1}\right)W_{2}+b_{2}$$

where $h_{n}$ is the input variable token and $W_{1}$, $W_{2}$, $b_{1}$, $b_{2}$ are the learnable parameters. The role of FFN is to extract more complex and abstract feature representations through a series of linear transformations and nonlinear activation functions. In wheat quality prediction, these feature representations can better capture the time-series characteristics of variables and the nonlinear relationships between variables.

In the context of time-series data, it is well established that each variable typically exhibits an implicit dependence structure and a nonlinear relationship. Conventional time-series methodologies frequently encounter challenges in explicitly modeling these intricate associations. To address these issues, this study constructs a heterogeneous graph structure based on physical driving mechanisms to explicitly model spatiotemporal interactions. Specifically, the iTransformer architecture is adopted to independently encode multidimensional time series as variable tokens, and the self-attention mechanism is used to capture long-range temporal dependencies within each variable. Each sequence is constructed as a node in a GCN network, such as temperature and humidity variables as graph nodes, and edge weights are defined based on the physical coupling relationships between variables. Through multi-layer GCN, information from neighboring nodes is aggregated to achieve feature propagation across variables.

To enhance the physical interpretability of the interactions between variables, the model constructs spatiotemporal association nodes through the following steps: In the GCN layer, node feature updates consider both temporal evolution and spatial interaction, using the temporal data encoding from iTransformer and preserving the original variable features through skip connections to avoid gradient vanishing. A trainable attention mechanism iteratively optimizes the adjacency matrix to capture data-driven variable dependencies. Consequently, this study proposes a graph structure to model the relationship between variables. Specifically, following the acquisition of the encoding between disparate variables through iTransformer processing, each sequence is constructed as a distinct node in the GCN network. Thereafter, the association nodes between variables are constructed based on the interaction between different variables. The construction of multivariate association nodes is achieved through the implementation of weighted pooling along the multivariate dimension. Subsequently, multi-head attention is applied to this matrix, resulting in the acquisition of vectors representing multi-variable correlation nodes by weighting and summing along the multi-variable dimension. Finally, these vectors are combined to form the overall feature representation of the multi-variable nodes. The feature vector of the variable correlation node is represented as follows:

$$\mathrm{multiVariable}\left(M\left(T\right),M\left(W\right)\right)=({\mathrm{ATT}}_{t}\left(M\left(T\right)\right),{\mathrm{ATT}}_{w}\left(M\left(W\right)\right))$$

where M(T) represents the embedding vector of the temperature variable, and matrix M(W) contains the embedding vector of the humidity variable. Here, ${\mathrm{ATT}}_{t}$ and ${\mathrm{ATT}}_{w}$ refer to the multi-head attention operations applied to the temperature and humidity variables, respectively.

In addressing the processing of the variable node-variable node, it is acknowledged that the interaction between variables is facilitated by establishing connections between each variable node, thereby enabling the variable association node to receive information from all other variable nodes. This process fosters the integration of multivariate information across diverse levels and effectively emulates long-distance dependencies among multivariate variables, thereby significantly reducing the complexity involved in identifying the dependencies between multivariate variables. In the course of processing variable features using GCN, we obtain variable association features through the embedding features of different variables. Subsequently, we input the variable association features and the features of the multivariate variables themselves into GCN. By generating and decoding graph information, we ultimately obtain the features of multivariate variables along with other influencing factors. The attributes of each variable node are directly defined by its multi-time-step observation data. For the input time series $X\in R^{T\times N}$, it is mapped to high-dimensional features $H\in R^{N\times D}$ through an embedding layer, where *T* is the length, *N* is the number of variables, and *D* is the embedding dimension. Based on the physical correlation between variables, the edge weights between nodes are dynamically constructed. The edge weights $e_{T-G}$ of the temperature and humidity nodes are calculated as follows:

(1) $$e_{T-G}=\frac{\mathrm{Cov}(T,G)}{\sigma_{T}\sigma_{G}}$$

where Cov is the covariance and σ is the standard deviation. The fully connected graph structure allows arbitrary variable nodes to interact directly, avoiding the loss of long-range correlation information due to local connections. Based on the definition of a heterogeneous graph $G=\left(V,E\right)$ (node set *V*, edge set *E*), a hierarchical graph convolution mechanism is used to capture cross-scale interaction features. For the feature initialization node *i*, the learning process of its hierarchical representation follows the rule of layer-wise propagation, and the embedding vector of layer *l* can be represented by the following formula:

$$h_{v_{i}}^{\left(l+1\right)}=\sigma \left(\sum_{j}\frac{1}{c_{ij}}h_{v_{j}}^{\left(l\right)}W^{\left(l\right)}+b^{\left(l\right)}\right)$$

σ is the nonlinear function ReLU, $W^{\left(l\right)}$ is the learning parameter of the *l* layer. $v_{i}$ refers to the node i in the Figure 4, $c_{ij}$ represents the normalization coefficient. The feature of each node in the Figure 4 is updated by the joint action of the features of other nodes.

$$h_{v_{i}}^{\left(l+1\right)}=h_{v_{i}}^{\left(l+1\right)}\left|\right|h_{v_{j}}^{\left(0\right)}$$

Among them, $h_{v_{j}}^{\left(0\right)}$ is the initial embedding of the node, which indicates the introduction of the jumping connection mechanism during the feature update process at the GCN layer. Here, || represents the feature splicing operation. In this way, the updated features of the current layer are directly spliced with the original input features, which not only retains the complete information of the original input but also allows the new features learned by the GCN layer to be combined with it, which helps to alleviate the gradient problem and enhance the richness of the feature representation.

Then, the output feature $h_{v_{j}}^{\left(l\right)}$ of each layer node i of GCN is combined along the column direction to obtain the final hidden state of the node. The combined column vector is linearly transformed using the learnable weight matrix $W_{g}$ to obtain the final hidden state $h_{vi}$ of the node i.

$$h_{vi}=W_{g}\left[h_{v_{i}}^{\left(0\right)};h_{v_{i}}^{\left(1\right)};\ldots ;h_{v_{i}}^{\left(L\right)}\right]$$

$$S_{i}=h_{vi}$$

Among them, $h_{v_{j}}^{\left(0\right)}$ is the initial embedding representation of the node *i*, and *L* is the number of GCN layers. Finally, the variable feature $S_{i}$ that fuses multivariate information and the information between multivariate variables can be obtained.

In order to coordinate the fusion process of the iTransformer encoding features and graph structure information, this model introduces a dynamic gating module (DGM) between the Transformer layer and the GCN layer, as illustrated in the circular node in Figure 4. This module dynamically adjusts the contribution weights of the two types of features through a learnable gating function, thereby avoiding feature conflicts caused by modal differences.

The Transformer output features are $H_{i}$, the initial node features of the GCN are $S_{i}$, and the dynamic gating values are generated by the interaction of the bimodal features:

$$\Gamma =\sigma \left(W_{\gamma }\left[H_{i}⊕S_{i}\right]+b_{\gamma }\right)$$

where ⊕ indicates element-by-element addition, σ· is the sigmoid function, and $W_{\gamma }$ is the learnable parameter. The fused features are obtained by gating the weights:

$$K_{i}=\Gamma ⊙H_{i}+\left(1-\Gamma \right)⊙S_{i}$$

Here, ⊙ is the element-by-element multiplication, $K_{i}$ is the final characteristic representation.

### 2.3. Model for Evaluating Wheat Quality Using K-Smeans

The classification of wheat quality is imperative for effective grain quality control and market pricing. Since wheat quality is influenced by multiple physical and chemical indicators, and these indicators undergo dynamic changes over time, the traditional K-means clustering algorithm has the following limitations in grain quality grading: initial cluster centers are randomly selected, making it prone to local optima; it relies solely on static data, ignoring the temporal evolution characteristics of quality indicators; and it is sensitive to noisy data, resulting in blurred cluster boundaries. Given the influence of wheat quality on multiple physicochemical indicators, which are dynamic over time, static methods of classification may overlook temporal information. To address this need, a novel classification method is proposed, underpinned by K-Smeans clustering. This approach integrates historical data with predictions from time-series models, leveraging temporal information to enhance the reliability and rationality of the clustering outcomes. Prior to the execution of the clustering analysis, the original wheat quality data must undergo preprocessing to ensure data consistency and validity. The proposed wheat quality index vector is defined as follows:

$$x_{t}=\left[\begin{array}{c} x_{t}^{\left(1\right)},x_{t}^{\left(2\right)},x_{t}^{\left(3\right)} \end{array}\right]$$

where $x_{t}^{\left(i\right)}$ represents the observed value of the *i* wheat quality indicator at time, there are three indicators in total. Since the wheat quality indicators may have different dimensions, the data need to be standardized so that they have the same scale. The Z-score normalization method is used:

$${\tilde{x}}_{t}^{\left(i\right)}=\frac{x_{t}^{\left(i\right)}-\mu_{i}}{\sigma_{i}}$$

Among them, $\mu_{i}$ and $\sigma_{i}$ are the mean and standard deviation of the *i* indicator, respectively. In addition, in order to make full use of time-series information, it is necessary to use the data from the past *P* time steps and the predicted data from the next *F* time steps as clustering features together. Let the future predicted value given by the time-series analysis model be

$${\hat{x}}_{t+f}=\left[{\hat{x}}_{t+f}^{\left(1\right)},{\hat{x}}_{t+f}^{\left(2\right)},{\hat{x}}_{t+f}^{\left(3\right)}\right],f=1,2,\ldots ,F$$

The sample evaluation index *G*, which is ultimately used for clustering, is constructed as follows:

(2) $$G_{t}=\left[x_{t-P},\ldots ,x_{t},{\hat{x}}_{t+1},\ldots ,{\hat{x}}_{t+F}\right]\in R^{(P+F+1)\times 3}$$

where $x_{t-P}$ represents the observed quality metrics (germination rate, fatty acid value, bulk density) over the past *P* time steps, and ${\hat{x}}_{t+1:t+F}$ denotes the model-predicted future data at time step *F*. This design enables the clustering process to simultaneously consider historical trends, current status, and future evolution, thereby enhancing the temporal consistency of the classification. As a result, each sample not only contains current quality information but also includes past trend information and future prediction trends, thereby improving the clustering’s adaptability to temporal dynamics. During the clustering iteration process, the model adjusts the weights of historical data and predicted data based on a time decay factor λ:

(3) $$w\left(\tau \right)=\frac{2}{1+e^{\lambda |\tau -t|}},\tau \in \left[t-P,t+F\right]$$

where τ represents the time step, and *t* is the current time. This function assigns higher weights to recent observations and gradually decreases the weights of future predictions, thereby balancing temporal reliability and forward-looking requirements. By examining multidimensional data, K-Smeans clustering can categorize wheat samples with analogous quality attributes into the same category, thereby establishing a foundation for subsequent quality grading. The K-Smeans clustering process is outlined as follows: Firstly, the wheat quality index data are standardized to ensure that each index has an equivalent scale. Secondly, historical data and future forecast data are integrated to construct a time-series feature of wheat quality indicators, thereby ensuring that the clustering analysis incorporates historical trends and future changes. Thirdly, the processed data are entered into the K-Smeans clustering algorithm to derive different wheat quality grades. Following the completion of K-Smeans clustering, each cluster thus obtained represents a distinct wheat quality grade. Through the analysis of the clustering results, researchers can identify the characteristics of the different clusters and subsequently classify wheat according to these characteristics. For instance, the specific quality grade of each cluster can be determined based on the numerical characteristics of the cluster center, and appropriate standards can be set for each grade based on the varying characteristics of wheat quality. This approach to analysis, founded on the principles of K-Smeans clustering, serves to enhance the scientific and objective nature of wheat quality classification. Moreover, by integrating time-series analysis with K-Smeans clustering, we are able to not only evaluate wheat quality based on historical data but also predict future quality trends, thereby facilitating more accurate classification and grading.

### 2.4. Model Evaluation Metrics

#### 2.4.1. Evaluation Methods for Predictive Models

In machine learning and data science research, building predictive models with good generalization ability is one of the core objectives. In order to systematically evaluate model performance and guide algorithm optimization, researchers need to establish a rigorous quantitative evaluation system. Among them, error metrics, as a basic tool for analyzing the prediction accuracy of models, play an irreplaceable role in key aspects such as deviation diagnosis, parameter tuning, and model selection. This paper theoretically discusses five commonly used error evaluation metrics in regression tasks: mean square error (MSE), root mean square error (RMSE), mean absolute error (MAE), mean absolute percentage error (MAPE), and symmetric mean absolute percentage error (SMAPE). By deriving their mathematical expressions, the theoretical characteristics of each metric are analyzed, and their applicable scenarios are discussed based on the characteristics of the error distribution.

As a basic error metric, MSE calculates the deviation between the predicted value and the true value using the second moment:

$$\mathrm{MSE}=\frac{1}{n}\sum_{i=1}^{n}{\left({\hat{y}}_{i}-y_{i}\right)}^{2}$$

To solve the dimensional problem of MSE, the RMSE with the same dimension as the original data is obtained by squaring:

$$\mathrm{RMSE}=\sqrt{\frac{1}{n}\sum_{i=1}^{n}{\left({\hat{y}}_{i}-y_{i}\right)}^{2}}$$

MAE uses the first-order moment to measure the prediction error:

$$\mathrm{MAE}=\frac{1}{n}\sum_{i=1}^{n}\left|{\hat{y}}_{i}-y_{i}\right|$$

MAPE provides a dimensionless evaluation scheme when cross-scale data comparison is required:

$$\mathrm{MAPE}=\frac{100\%}{n}\sum_{i=1}^{n}\left|\frac{{\hat{y}}_{i}-y_{i}}{y_{i}}\right|$$

This indicator is widely used in business scenarios such as demand forecasting and sales forecasting. However, when the true value approaches zero, the denominator singularity problem occurs. To this end, SMAPE improves the calculation stability through symmetrical processing:

$$\mathrm{SMAPE}=\frac{200\%}{n}\sum_{i=1}^{n}\frac{|{\hat{y}}_{i}-y_{i}|}{|{\hat{y}}_{i}\left|+\right|y_{i}|}$$

#### 2.4.2. Evaluation Methods for Cluster Models

In order to better classify the quality of wheat, this model uses the silhouette coefficient, Davies–Bouldin index, and Dunn index to evaluate the clustering model so as to obtain the best clustering results and complete the grade planning of wheat. Among them, the silhouette coefficient comprehensively evaluates the rationality of the clustering structure by quantifying the closeness of the sample to its cluster and its separation from other clusters. For a single sample i, its silhouette coefficient is defined as follows:

$$s\left(i\right)=\frac{b(i)-a(i)}{max\{a(i),b(i)\}}$$

Among them, a(i) represents the average distance from sample i to other samples in the same cluster, reflecting the tightness within the cluster; b(i) is the average distance from sample *i* to all samples in the nearest cluster, representing the degree of separation between clusters. The overall silhouette coefficient is the arithmetic mean of all samples s(i):

$$\mathrm{Silhouette}\;\mathrm{Score}=\frac{1}{n}\sum_{i=1}^{n}s\left(i\right)$$

The value range is [−1,1], and the closer the value is to 1, the better the clustering structure. This index is applicable to any distance metric space, but in high-dimensional data, it may lead to distorted evaluation due to “dimensional disaster”.

The Davies–Bouldin index evaluates the tightness of clustering by calculating the inter-cluster similarity. It is defined as follows:

$$\mathrm{DB}=\frac{1}{k}\sum_{i=1}^{k}max_{j\neq i}\left(\frac{S_{i}+S_{j}}{d(c_{i},c_{j})}\right)$$

where $S_{i}$ represents the average distance from the center of mass $c_{i}$ to the samples in cluster $C_{i}$, and $d(c_{i},c_{j})$ is the distance between the cluster centers of mass. The value range of this indicator is (0,+∞], and a smaller value indicates higher clustering quality. It is sensitive to noisy data and requires that the distance metric satisfy the triangle inequality.

The Dunn index calculates the shortest distance between any two cluster elements (between classes) divided by the maximum distance within any one cluster (within class). It is calculated as follows:

$$DI=\frac{\min_{1\leq k<k\prime \leq m}d_{min}\left(C_{k},C_{k\prime }\right)}{\max_{1\leq l\prime \leq m}diam\left(C_{l\prime }\right)}$$

$$d_{min}\left(C_{k},C_{k^{\prime }}\right)=\min_{x\in C_{k},x^{\prime }\in C_{l^{\prime }}}dist\left(x,x^{\prime }\right)$$

$$diam\left(C_{l}\prime \right)=\max_{1\leq i<j\leq |C_{l}\prime |}dist\left(x_{i},x_{j}\right)$$

The larger the value, the greater the distance between classes and the smaller the distance within a class. It means that any two clusters are selected, and a sample point is assigned from each cluster to calculate the distance, and the minimum distance is taken as the distance between the two clusters. $diam\left(C_{l\prime }\right)$ means that any cluster is selected, and the distance between any two points in the cluster is calculated, and the maximum distance is taken as the diameter of the circle covering all points in the cluster. Note that this circle diameter is not the minimum circle diameter that can cover all points in the cluster, because the complexity of solving the minimum circle covering problem is too high.

## 3. Results and Discussion

### 3.1. Comparison of Predictive Models

In this section, in order to effectively evaluate the performance of SGCNiFormer in predicting the changes in various quality evaluation indicators during wheat storage, seven recognized prediction models are selected as benchmarks, including Autoformer [35], Crossformer [36], TimesNet [37], TiDE [38], FEDformer [39], Stationary [40], and PatchTST [41]. With the same training set, test set, and validation set, each model is tested, and its prediction error is analyzed. To accurately and objectively evaluate the performance of the above models, we used five evaluation metrics from time-series analysis to calculate the error value of each model, including MSE, RMSE, MSPE, MAPE, and MAE.

The model was built using the open-source deep learning framework PyTorch, and experiments were run using NVIDIA 4090 GPUs. The time-series experiment settings were as follows: batch size of 32, learning rate of 0.0001, number of GCN layers of three, number of epochs of 20, and Adam as the optimizer.

**Figure 5:**
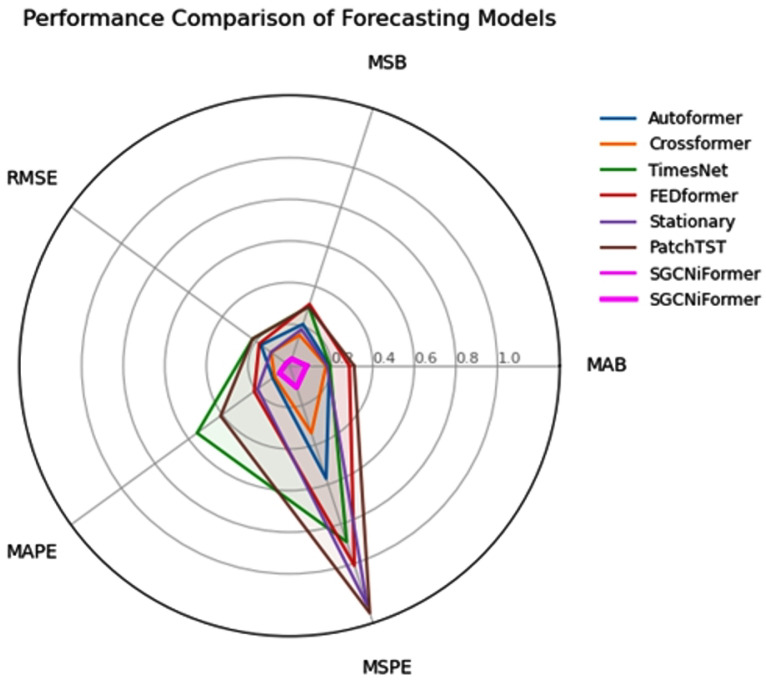
Comparative experiment of time-series analysis.

In this paper, we propose the SGCNiFormer model and validate its superiority through comparative experiments. The table above lists the performance of SGCNiFormer and other mainstream models on multiple evaluation metrics, as shown in Table 2. First, from the metrics of MAE, MSE, and RMSE, SGCNiFormer performs excellently in all metrics, especially in MAE and MSE, with values of 0.082 and 0.035, respectively, significantly better than other comparison models. This result indicates that SGCNiFormer has significantly improved the accuracy of time-series prediction. As shown in Figure 5, this study compared the performance of various time-series prediction models through radar charts. All indicators are indicated as having better performance with smaller values. The SGCNiFormer model shows significant advantages in all indicators. Its radar map polygon area is the smallest and closest to the coordinate origin, indicating that its prediction error is comprehensively lower than that of the baseline model.

The superior performance of SGCNiFormer is primarily attributed to its innovative integration of the advantages of graph convolutional networks (GCN) and the Transformer architecture. Specifically, the GCN module introduces a graph neural network (GNN) structure, enabling the model to deeply mine the complex relationships between nodes in time-series data, especially in scenarios with strong nonlinearity and multivariate dependencies. GCN effectively captures local and global dependencies between nodes through multi-layer graph convolution operations, thereby enhancing the model’s ability to learn complex time-dependent patterns. This capability is particularly important when handling multi-variable complex time-series data, as traditional methods often struggle to directly model such high-dimensional dependency structures. Compared with traditional time-series models, GCN has the advantage of automatically establishing dependencies between nodes through graph convolution without the need for manually setting specific dependency structures. Through this mechanism, SGCNiFormer can more accurately capture the potential spatio-temporal dependency patterns in the data, which are particularly critical for complex time-series prediction, especially in scenarios where long-term interactions between different variables need to be considered. Additionally, the multi-head self-attention mechanism in the Transformer architecture further enhances the performance of SGCNiFormer. The Transformer module efficiently handles long-range dependencies, while the multi-head self-attention mechanism enables the model to compute attention weights in parallel across different subspaces, thereby capturing multi-level dependencies in time-series data. Combined with the graph structure of GCN, SGCNiFormer performs well in processing long time-series data, avoiding the limitations of traditional models in modeling long-term dependencies.

**Figure 6:**
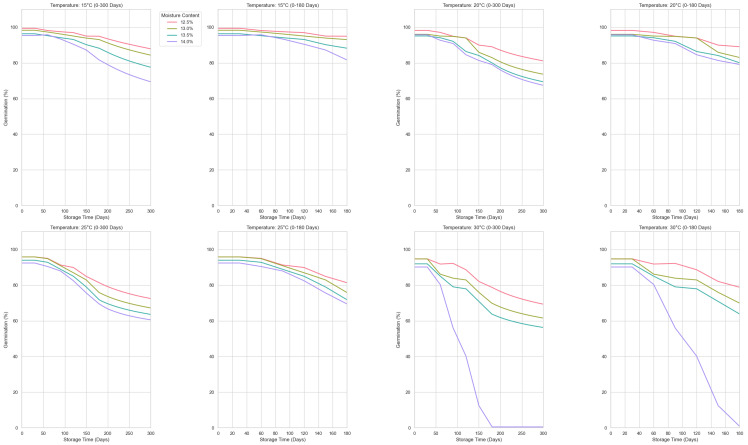
Comparison of wheat germination rate experimental data and predicted data.

By combining the advantages of GCN and Transformer, SGCNiFormer not only effectively captures complex dependencies in time-series data but also globally handles dependencies over long time spans, further improving the prediction accuracy of the model. When compared with other advanced time-series prediction models (such as PatchTST, FEDformer, TimesNet, etc.), SGCNiFormer performs outstandingly in MAPE and MSPE, with an MAPE of 0.06 and an MSPE of 0.11, far lower than other models, proving its superiority in complex time series prediction tasks. This finding indicates that SGCNiFormer not only effectively reduces errors but also provides more reliable prediction results when dealing with complex and diverse real-world applications. Figure 6 shows the changes in germination rate at different storage temperatures. The first and third columns are the output results of the prediction model, and the second and fourth columns are the line graphs of the results collected every thirty days in the experimental data. Specifically, under all test conditions, the model successfully predicted the variation of the germination rate with storage time and accurately reflected the differences under different humidity levels. It can be seen from this that the SGCNiFormer model shows high accuracy, especially in effectively predicting the changing trend of wheat germination rate under different storage temperatures and humidity conditions.

In summary, SGCNiFormer innovatively integrates graph convolutional networks and the Transformer architecture, particularly leveraging GCN’s ability to capture complex relationships in time-series data, enabling it to achieve significant advantages across multiple key evaluation metrics. This demonstrates its potential and application value in complex time-series prediction tasks. Future work can focus on further optimizing the model structure to enhance its performance on large-scale datasets and in real-time prediction scenarios.

### 3.2. Ablation Experiments of Predictive Models

In the SGCNiFormer model, the roles of each module and their impact on performance can be systematically analyzed through ablation experiment results, as shown in Table 3. First, the GCN module plays a decisive role in modeling the spatial dependency between environmental variables and wheat quality indicators. Comparing Model 1 and Model 2, the introduction of GCN reduces MAE from 0.12 to 0.09, MSE from 0.091 to 0.032, and RMSE from 0.12 to 0.07. This significant improvement indicates that GCN captures the nonlinear interaction characteristics between temperature, humidity, and quality indicators through graph structure, effectively resolving the topological associations in multidimensional data, and thus becoming the core driver of model performance.

Second, the Dynamic Gate Module (DGM) further optimizes model performance by adaptively integrating features from GCN and Transformer. Compared with Model 2 and the complete model SGCNiFormer, the RMSE decreases from 0.07 to 0.02, and the MAPE decreases from 0.11 to 0.06 after adding DGM. DGM dynamically coordinates the weight allocation of spatial and temporal features through a gating mechanism. For example, in long-term trend prediction, it emphasizes the global temporal modeling capabilities of the Transformer, while in environmental variable coupling analysis, it reinforces the spatial interaction effects of the GCN, thereby avoiding modal conflicts and improving generalization capabilities. Although Model 2 MSE is slightly lower than that of SGCNiFormer, the significant optimization of RMSE and MAPE highlights the necessity of DGM in complex scenarios.

Finally, iTransformer provides basic temporal encoding capabilities as the backbone network. Model 1’s MAE and MAPE are significantly higher than other models, indicating that temporal encoding alone is insufficient to fully model the complexity of dynamic systems. However, when combined with GCN, its performance improves significantly, demonstrating a synergistic effect between iTransformer’s attention mechanism and GCN’s spatial modeling capabilities. For example, iTransformer captures the nonlinear decay trend of germination rate, while GCN analyzes the modulating effect of environmental variables on this trend, and the two complement each other to enhance the model’s overall prediction capability.

### 3.3. Comparison of Clustering Models

In this study, we propose the K-Smeans clustering algorithm for the classification of wheat quality, taking into account three quality evaluation indicators: germination rate, fatty acid value, and weight per unit area. These indicators are derived from physiological, chemical, and physical properties.As the sampled values of each index are obtained at different times, this paper proposes an efficient time prediction model, SGCNiFormer, to supplement the sample information of each index value. The results demonstrate that the prediction is within a reasonable range. Consequently, the K-Smeans clustering algorithm incorporates temporal data to enhance the stability and rationality of the clustering process. This study further investigates the merits of K-Smeans in wheat quality clustering by contrasting it with the K-means++ clustering algorithm. During the clustering analysis, the clustering results with different numbers of clusters are evaluated using three different clustering evaluation metrics: the silhouette coefficient, the Davies–Bouldin index (DB index), and the Dunn index. The optimal number of clusters is determined based on these results.

**Figure 7:**
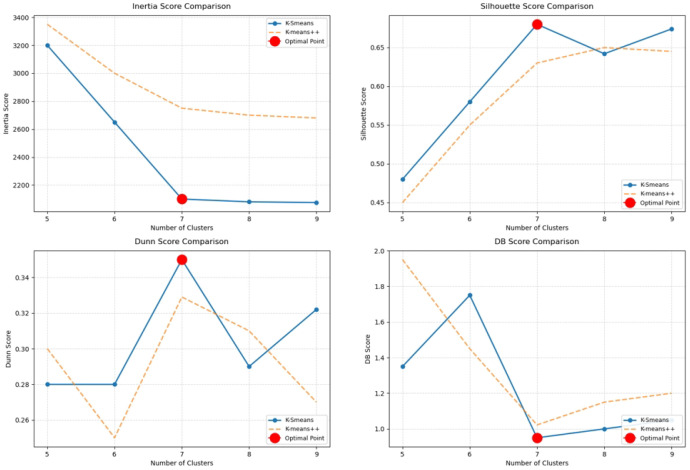
Evaluation coefficient diagram of K-Smeans clustering.

**Figure 8:**
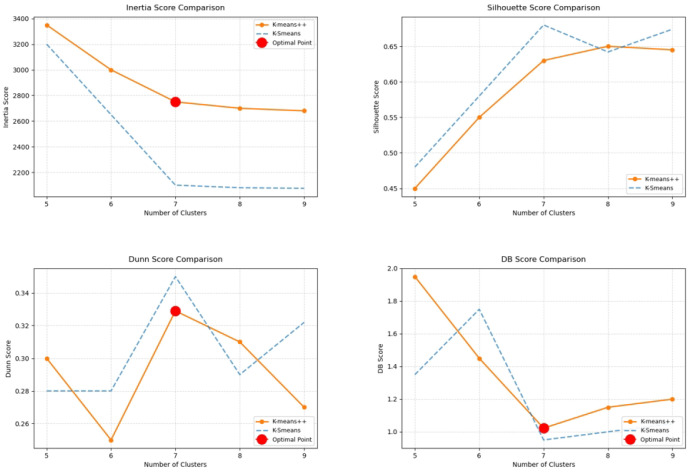
Evaluation coefficient plot for K-means++ clustering.

As demonstrated in Figure 7 and Figure 8, the silhouette score of K-Smeans clustering attains its maximum value when the number of clusters is 7, signifying that the similarity of the samples within a cluster is high at this point, while the dissimilarity between clusters is substantial. The DB index and Dunn index, on the other hand, demonstrate the minimum and maximum values, respectively, thereby validating the conclusion that the clustering outcome is optimal when the number of clusters is 7. Consequently, based on the evaluation indices of the clustering process, it can be concluded that the optimal number of clusters for K-Smeans clustering is 7. However, in contrast, the K-means++ algorithm, despite exhibiting a marginally elevated silhouette coefficient when the number of clusters is 7, demonstrates suboptimal performance in both the DB index and the Dunn index. This suggests that K-means++ tends to exhibit significant overlap between clusters, resulting in a suboptimal clustering outcome. The underlying cause of this phenomenon is that the initial cluster center selection of the K-means++ algorithm relies on a random process, which is prone to local optima and thus affects the accuracy and stability of the clustering results. K-Smeans avoids this problem by selecting the farthest data point as the cluster center during initialization, further enhancing the stability and accuracy of clustering.

A comparison of the two algorithms reveals that K-Smeans offers significant advantages in the analysis of wheat quality. First, the K-Smeans algorithm can reasonably classify samples into different quality grades based on historical and future predicted data of wheat quality, thereby improving the stability and accuracy of the clustering results. Second, the K-Smeans algorithm improves the effectiveness of the clustering results by optimizing the selection of the initial cluster center, avoiding the instability of the traditional K-means algorithm in the selection of the initial cluster center. This enhances the adaptability of K-Smeans to the dynamic characteristics of data, particularly in the context of complex datasets such as wheat quality classification, thereby providing a more scientific and objective approach to evaluating wheat quality grades.

The K-Smeans algorithm boasts significant advantages in the context of cluster analysis of wheat quality indicators, particularly when the number of clusters is set at seven. In such instances, all evaluation indicators attain their optimal values, signifying that the clustering results are the most rational at that particular juncture. The K-Smeans algorithm facilitates more effective classification of wheat samples into distinct quality grades, thereby providing a scientific foundation for the assessment and classification of wheat quality. This approach enables not only the scientific evaluation of historical data but also the incorporation of future predicted trends, thereby providing more reliable support for the accurate classification of wheat quality.

### 3.4. Analysis of Results

The integration of time-series prediction and K-Smeans clustering analysis has led to substantial enhancements in the management of wheat quality, particularly with regard to preserving its stability during storage. Conventional quality assessment methods frequently depend on static data and fail to consider the temporal variations in wheat quality, which can result in errors or inaccuracies in quality management. To address these limitations, a novel clustering analysis method is proposed, underpinned by the K-Smeans algorithm. This approach not only utilizes historical data but also incorporates the prediction of future data, thereby enhancing the adaptability of clustering analysis to the time-dynamic changes in wheat quality.

First, time-series prediction provides critical time-dimension information for clustering analysis. In conventional clustering methods, data are typically static, precluding the analysis of changes in wheat quality over time. However, with a time-series prediction model, we can forecast the probable trend of wheat quality over time. Specifically, we constructed a time-series model based on historical data to predict wheat quality at a future point in time. These future predictions were then integrated with historical data to inform the clustering analysis, ensuring that the quality characteristics of each sample reflected not only the present state but also past changes and future trends. This approach enhanced the comprehensiveness of the clustering process, enabling more precise capture of the dynamic changes in wheat quality. Consequently, this methodology facilitates the identification and prediction of fluctuations in wheat quality during storage and subsequently enables the implementation of appropriate measures to control and maintain wheat quality.

Secondly, the employment of the K-Smeans clustering algorithm in this study serves to further optimize the shortcomings of the conventional K-means algorithm. The K-means algorithm is highly sensitive to the selection of the initial cluster center, which frequently leads to the discovery of local optima. These local optima have the potential to adversely impact the accuracy and stability of the clustering process. To address this issue, the K-Smeans algorithm utilizes a more effective method for selecting the initial cluster center. Specifically, K-Smeans selects the data point that is farthest from the selected cluster center as the new cluster center during initialization. This strategy effectively avoids the common problem of improper selection of initial cluster centers in the K-means algorithm, ensuring the stability and accuracy of the clustering results. Through this optimization, K-Smeans can better cluster different wheat qualities, thereby providing a more scientific basis for quality classification in wheat storage, as shown in Table 4 and Table 5.

The integration of time-series prediction with K-Smeans cluster analysis enables the accurate assessment of current wheat quality and the prediction of future quality changes, facilitating the implementation of suitable storage management strategies. Specifically, storage managers can utilize the outcomes of cluster analysis to comprehensively understand the quality characteristics of each cluster, enabling the implementation of targeted measures based on these characteristics. For instance, for wheat of lower quality, specific preservation measures can be implemented, such as reducing storage temperature or adjusting humidity levels, to decelerate the rate of quality decline. Conversely, for wheat of higher quality, conventional storage conditions can be employed to minimize unnecessary expenses. Additionally, through time-series prediction, storage managers can ascertain the timing of potential quality fluctuations, enabling proactive preparation and mitigation of losses incurred by sudden quality declines.

However, this method also has certain limitations. In practical applications, data quality significantly impacts the accuracy of prediction results. Wheat quality is influenced by various factors, such as storage conditions and climate changes. If data collection contains errors or omissions, this may affect the model’s predictive accuracy. Additionally, the model’s generalization ability may face challenges when applied to different crop types or storage environments. Although this method demonstrates excellent performance in wheat storage management, its applicability to other crops or environments requires further validation.

The integration of time-series prediction with K-S means clustering analysis empowers wheat storage management to not only ensure the stability of wheat quality but also more effectively classify and manage wheat of varying qualities. Leveraging historical data and predictive information, managers can scientifically adjust storage strategies to ensure that wheat maintains its optimal quality during storage, thereby mitigating market risks associated with quality fluctuations. This approach enhances the efficacy of wheat storage management and provides a novel technical framework for the long-term storage and quality control of agricultural products.

## 4. Conclusions

The present study endeavors to address the scientific challenge of dynamic prediction and evaluation of wheat quality during storage by establishing a data-driven intelligent decision-making system. The findings suggest that conventional methodologies possess inherent constraints when it comes to the analysis of the spatiotemporal evolution mechanisms of wheat quality. In contrast, the SGCNiFormer model, an integration of graph convolutional networks and dynamic gating, has been demonstrated to achieve a substantial enhancement in long-term prediction accuracy. An enhanced K-means dynamic grading algorithm was developed to construct a quality assessment system adaptable to changes in storage environments. The system exhibited both excellent prediction accuracy and assessment consistency in the experiments, thereby providing a scientific basis for intelligent grain storage management. By optimizing quality prediction and grading assessment, the system can significantly improve the precision of management during storage, demonstrating significant practical application potential. Subsequent research endeavors will further expand the model’s applicability, explore its application in different agricultural products, and investigate potential improvements in real-time data processing and dynamic decision-making to drive the continuous development of intelligent storage systems.

## Figures and Tables

**Table 1:** The equilibrium of wheat at different temperatures and moisture levels is expressed as a percentage of moisture.

Moisture Content/%	Temperature/℃			
	**15**	**20**	**25**	**30**
12.5	56	58	60	62
13	60	61	63	65
13.5	63	65	66	68
14	67	69	71	73

**Table 2:** Compare the radar chart of the experiment.

Model	MAE	MSE	RMSE	MAPE	MSPE
Autoformer	0.192	0.210	0.17	0.10	0.57
Crossformer	0.176	0.155	0.11	0.09	0.34
TimesNet	0.194	0.299	0.22	0.55	0.89
FEDformer	0.288	0.311	0.18	0.21	1.01
Stationary	0.181	0.184	0.11	0.19	1.21
PatchTST	0.311	0.298	0.22	0.41	1.25
SGCNiFormer	0.082	0.035	0.02	0.06	0.11

**Table 3:** Ablation experiment of time-series analysis.

Model	MAE	MSE	RMSE	MAPE	MSPE
**Model 1**	0.12	0.091	0.12	0.19	0.29
**Model 2**	0.09	0.032	0.07	0.11	0.30
**SGCNiFormer**	0.082	0.035	0.02	0.06	0.11

Model 1: Removed the GCN module and the dynamic gate module and used the iTransformer encoding. Model 2: Removed the dynamic gate module, used iTransformer encoding, and used GCN to obtain features of the interactions between different variables.

**Table 4:** Range of each indicator for each grade of wheat.

Level	density_min	density_max	fatty_acid_min	fatty_acid_max	germination_min	germination
0	792.4923	800.8472	10.90917	15.35083	80.26147	87.49203
1	731.5515	734.1664	43.14179	52.3	0.840336	22.63305
2	800.8472	804.3139	8.8807	10.90917	87.49203	93.82123
3	734.1664	749.4499	33.22769	43.09385	23.55742	47.5817
4	804.3139	812.3183	2.135091	8.8807	93.82123	99.43802
5	771.2496	792.4923	15.35083	20.15644	63.70693	80.26147
6	750.3385	770.708	20.15644	32.58462	48.10924	63.70693

**Table 5:** Quantity of wheat of each grade in the cluster.

Cluster	Count
0	574
1	36
2	211
3	1139
4	640
5	28
6	28

## Data Availability

The raw data supporting the conclusions of this article will be made available by the authors on request.
